# Deciphering common and specific transcriptional immune responses in pea towards the oomycete pathogens *Aphanomyces euteiches* and *Phytophthora pisi*

**DOI:** 10.1186/s12864-015-1829-1

**Published:** 2015-08-21

**Authors:** Sara Hosseini, Malin Elfstrand, Fredrik Heyman, Dan Funck Jensen, Magnus Karlsson

**Affiliations:** Department of Forest Mycology and Plant Pathology, Uppsala BioCenter, Swedish University of Agricultural Sciences, Box 7026, SE-75007 Uppsala, Sweden

**Keywords:** Hormonal signalling, Microarray, Phenylpropanoid pathway, *Pisum sativum*, Plant immunity, Resistance gene, Transcriptional response

## Abstract

**Background:**

Root rot caused by *Aphanomyces euteiches* is one of the most destructive pea diseases while a distantly related species *P. pisi* has been recently described as the agent of pea and faba bean root rot. These two oomycete pathogens with different pathogenicity factor repertories have both evolved specific mechanisms to infect pea. However, little is known about the genes and mechanisms of defence against these pathogens in pea. In the present study, the transcriptomic response of pea to these two pathogens was investigated at two time points during early phase of infection using a *Medicago truncatula* microarray.

**Results:**

Of the 37,976 genes analysed, 574 and 817 were differentially expressed in response to *A. euteiches* at 6 hpi and 20 hpi, respectively, while 544 and 611 genes were differentially regulated against *P. pisi* at 6 hpi and 20 hpi, respectively. Differentially expressed genes associated with plant immunity responses were involved in cell wall reinforcement, hormonal signalling and phenylpropanoid metabolism. Activation of cell wall modification, regulation of jasmonic acid biosynthesis and induction of ethylene signalling pathway were among the common transcriptional responses to both of these oomycetes. However, induction of chalcone synthesis and the auxin pathway were specific transcriptional changes against *A. euteiches*.

**Conclusions:**

Our results demonstrate a global view of differentially expressed pea genes during compatible interactions with *P. pisi* and *A. euteiches* at an early phase of infection. The results suggest that distinct signalling pathways are triggered in pea by these two pathogens that lead to common and specific immune mechanisms in response to these two oomycetes. The generated knowledge may eventually be used in breeding pea varieties with resistance against root rot disease.

**Electronic supplementary material:**

The online version of this article (doi:10.1186/s12864-015-1829-1) contains supplementary material, which is available to authorized users.

## Background

Legumes are important sources of proteins for human food and animal feed. In addition, legumes improve soil fertility and decrease the need for N fertilizers through symbiotic interactions with nitrogen fixing bacteria and thus contribute to the sustainability of agriculture [1]. Field pea, *Pisum sativum*, is a legume crop that is grown on over 25 million acres worldwide and in Europe dry pea production is the highest within legume production. The main threat to pea yields are diseases, including *Aphanomyces euteiches* [2] that causes seedling damping off and root rot disease of many legumes. It is considered as the most devastating pea pathogen, causing up to 80 % losses each year [3, 4]. It is widespread in North America, Europe, Japan, Australia and New Zealand [5, 6]. *Phytophthora pisi* is a recently described species, which causes root rot on pea and faba bean (*Vicia faba*), and is capable of infecting certain other legumes closely related to these crops [7]. As an emerging pathogen, it represents a potential threat for pea cultivation.

Both these pathogens are oomycetes, which belong to the kingdom Stramenopila and are evolutionary related to brown algae. In the asexual stage bi-flagellated motile zoospores are released in the soil, swim chemotactically towards the plant roots, encyst at the root surface, germinate and subsequently infect the host roots. In the sexual cycle, thick-wall oospores are formed that survive in the soil for many years as the primary source of inoculum. Efficient chemical control of the pea root rot diseases caused by *A. euteiches* and *P. pisi* is not available and crop rotation and other cultural practices remain the only solutions to avoid the disease. Although the use of resistant pea varieties would be the most economical and ecological strategy to control the disease, no resistant variety to either pathogen is commercially available so far.

*Pisum sativum* has a large and complex genome and currently few comprehensive genomic resources exist. The lack of a sequenced genome is a limiting factor for molecular and –omics approaches for research on this plant [8]. However, the advantage of knowledge and tools available for the legume model species *Medicago truncatula* are used for research on pea [9–11] since high level of genetic homology and synteny between these two species are reported [12, 13]. A *M. truncatula* microarray was recently used successfully to study the transcriptomic response of pea during infection by *Mycosphaerella pinodes* [14].

Our present understanding of the early molecular interactions between *A. euteiches* or *P. pisi* and pea are very limited. Plant-pathogen interaction is viewed as a multi-layered process, where in the first layer of the defence system plants can recognize conserved microbe- or pathogen-associated molecular patterns (MAMPs or PAMPs) and initiate pattern-triggered immunity (PTI) [15]. Specialized pathogens secrete proteins called effectors that suppress PTI and result in effector-triggered susceptibility (ETS). Subsequently, certain plants recognize particular effectors, or their activity, by resistance (R) proteins, which lead to activation of the second layer of defence, effector-triggered immunity (ETI) [16, 17]. ETI is stronger in amplitude than PTI and can involve the same or different signalling sectors than PTI, but both layers involve massive changes of gene activity and extensive reprogramming of the cell metabolism.

For a successful defence the activation of plant responses must be rapid, efficient and targeted. It is shown that the signalling sectors defined by the phytohormones salicylic acid (SA), jasmonic acid (JA) and ethylene (ET) are important in plant immunity [18]. SA is generally involved in immunity against biotrophic and hemi-biotrophic pathogens, while the JA and ET sectors are involved in immunity against necrotrophic pathogens and herbivorous insects. Other phytohormones such as abscisic acid, auxin and gibberellins are also involved in plant immune signalling [19]. In addition, a number of phenylpropanoid compounds with antimicrobial activity have been shown to restrict pathogen growth [20, 21]. In legumes, flavonoid compounds that are crucial in the initiation of symbiotic interactions with rhizobia also play a role as defence compounds and as signalling molecules [22].

*Aphanomyces* spp. belongs to Saprolegniales and includes numerous destructive plant and animal pathogens, whereas *Phytophthora* spp. belongs to Peronosporales and includes species only pathogenic to plants. This diversity within the oomycetes reflects different evolutionary histories and different mechanisms of infection between Saprolegniales and Peronosporales [23]. One major difference between *Aphanomyces* and *Phytophthora* is depicted in their effector repertoires, where *Phytophthora* species contain a large spectrum of RXLR effectors while no RXLR genes are identified in the *A. euteiches* genome [24]. Despite being distantly related species, *A. euteiches* and *P. pisi* have both evolved specific pathogenicity on pea as a common host. Therefore, we hypothesize that *A. euteiches* and *P. pisi* manipulate the PTI or ETI defence signalling in pea in common and distinct ways depending on differences in their effector repertories, which subsequently leads to activation of common or distinct pea defence responses.

The aim of this project was to study the transcriptomic response of pea plants towards *A. euteiches* and *P. pisi* during the initial phase of infection, using heterologous probing on a *M. truncatula* microarray. We aim to identify differentially regulated genes during early infection, to make inferences about pea immune-related pathways that are commonly or specifically regulated during interaction with these two oomycetes pathogens.

## Results

### Infection process evaluation

The infection process of pea roots with *A. euteiches* and *P. pisi* was evaluated by analysing gene expression of selected defence marker genes such as *ACO* (*1-aminocyclopropane-1-carboxylate oxidase*), *Pi49* (*PR10-like*), *ABA17* (*abscisic acid responsive gene*) and *chit4* (*chitinase 4*), using reverse transcription quantitative PCR (RT-qPCR). Relative expression of all genes increased with time during infection (Additional file 1). Expression of *ACO*, *ABA17* and *Pi49* were significantly (*P* ≤ 0.05) induced during *A. euteiches* infection from 20 h post inoculation (hpi) and onwards, and at 48 hpi during *P. pisi* infection. Infection samples at 6 and 20 hpi were selected to study the early global transcriptomic response of pea to *A. euteiches* and *P. pisi*, using microarray technology.

### Microarray analysis

#### Number of differentially expressed genes

**Fig. 1:**
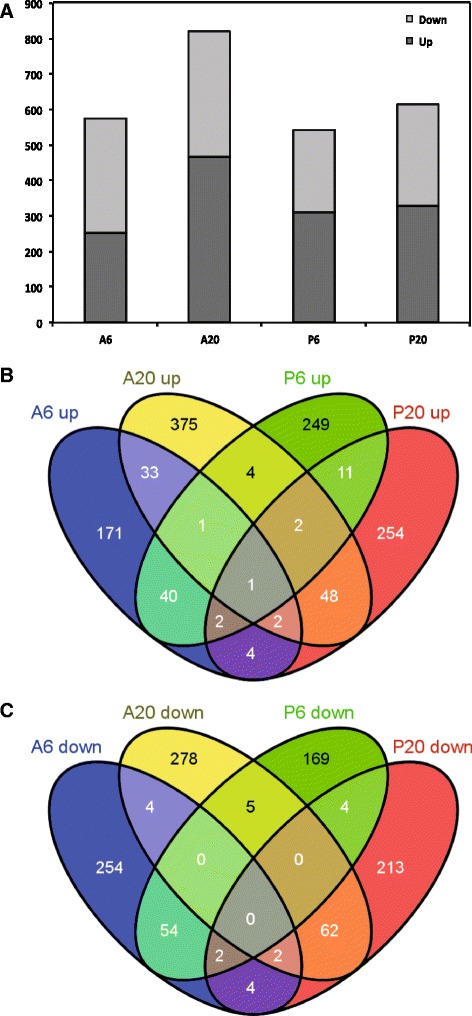
Number of differentially expressed pea genes during interaction with *A. euteiches* and *P. pisi*, and the overlap between time points and species. **a** Overview of the number of significantly up regulated (dark grey) and down regulated (light grey) genes compared to the control samples at each time point of infection. The overlap between (**b**) up regulated and (**c**) down regulated gene sets

All 40425 probe sequences (37976 genes) included on the microarray showed an analysable signal. Applying the linear models for microarray data (Limma model) [25], 2179 and 3193 genes were identified as responsive to *A. euteiches* at 6 hpi and 20 hpi, respectively, while 1610 and 1826 genes were found as responsive to *P. pisi* at 6 hpi and 20 hpi, respectively (*P* ≤ 0.05) (Table 1, Additional files 2 and 3). These lists of responsive genes to each treatment were used for analysis of biochemical pathways in the Kyoto Encyclopedia of Genes and Genomes (KEGG) database. In order to focus the analysis on the genes with higher fold change expression compared to the control samples we considered a cut off (≥0.584) on the log_2_ ratio fold change expression treatment/control of the genes with *P* ≤ 0.05. Therefore, genes with ≥ 1.5 fold induction and ≤ 0.67 fold suppression compared to control samples in the same time point were defined as differentially expressed genes. In response to *A. euteiches,* 574 and 817 genes were differentially expressed (*P* ≤ 0.05 and log_2_ ratio ≥ 0.584) at 6 hpi and 20 hpi, respectively (Table 1, Fig. 1a, Additional file 2). In response to *P. pisi*, 544 and 611 sequences were differentially expressed (*P* ≤ 0.05 and log_2_ ratio ≥ 0.584) at 6 hpi and 20 hpi, respectively, compared to the control samples at the same time point (Table 1, Fig. 1a, Additional file 3). All differentially expressed genes were associated with their respective Gene Ontology (GO) terms and clustered based on their GO terms. Sixty-four percent of all differentially expressed genes were successfully associated with a GO number and categorized across the three main GO categories of biological process, cellular component and molecular function (Additional file 4). The remaining 36 % represented sequences of currently unknown functions.

**Table 1:** Number of genes related to each treatment in response to *Aphanomyces euteiches* and *Phytophthora pisi* at 6 hpi and 20 hpi

Gene sets	*A. euteiches* 6 hpi		*A. euteiches* 20 hpi		*P. pisi* 6 hpi		*P. pisi* 20 hpi	
	Up^a^	Down^a^	Up	Down	Up	Down	Up	Down
Genes responsive to the treatment^b^	1120	1059	1506	1687	797	813	919	907
Differentially expressed genes^c^	254	320	466	351	310	234	324	287

^a^Up and Down refers to the up regulated and down regulated genes, respectively

^b^Refers to the genes with a *P* value ≤ 0.05 in response to the pathogen compared to the control samples at the same time point

^c^Refers to the genes with a *P* value ≤ 0.05 and the log_2_ ratio ≥ 0.584 (>1.5 fold induction or ≤ 0.67 repression) compared to the control samples at the same time point

#### Distinct transcriptional plant responses are featured during the early phase of infections by *P. pisi* and *A. euteiches*

The overlap of differentially expressed genes in response to *A. euteiches* and *P. pisi* over time was determined (Fig. 1b, c). Only a limited number of common genes were up regulated or down regulated in response to the two pathogens at 6 and 20 hpi. In contrast, large sets of genes were uniquely regulated in response to each pathogen and at each time point.

**Fig. 2:**
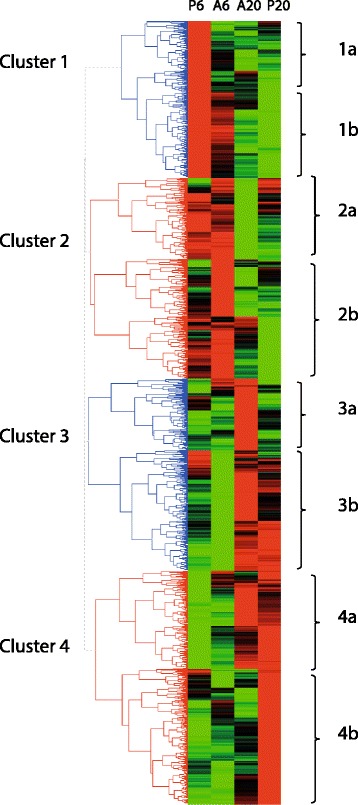
Clustering of differentially expressed pea genes in response to *A. euteiches* and *P. pisi.* Hierarchical clustering of all differentially expressed genes (*P* ≤ 0.05, ≥ 1.5 fold induction or ≤ 0.67 fold repression) at 6 hpi and 20 hpi compared to the mock-inoculated control samples generated by HCE3.5 software with the complete linkage method and the Manhattan distance measure. Red and green represent up regulated and down regulated genes, respectively. Four classes of genes were defined according to their expression profiles. Cluster 1 and 4 corresponds to genes highly up regulated at 6 hpi and 20 hpi in response to *P. pisi* (P6 and P20, respectively), while cluster 2 and 3 corresponds to genes up regulated at 6 hpi and 20 hpi in response to *A. euteiches* (A6 and A20, respectively). Each cluster was urther divided into smaller sub-clusters containing genes with common regulatory patterns. Sub-clusters 1b and 2a included genes induced in response to both pathogens at 6 hpi, while sub-clusters 1a and 2b included genes specifically induced in response to *P. pisi* and *A. euteiches* at 6 hpi, respectively. Sub-clusters 3b and 4a included genes induced in response to both pathogens at 20 hpi, while sub-clusters 3a and 4b represented genes specifically induced at 20 hpi in response to *A. euteiches* and *P. pisi*, respectively

Furthermore, hierarchical clustering was performed for genes differentially regulated in at least one interaction. Four major clusters could be distinguished (Fig. 2). Clusters 1 and 2 represented genes mainly induced at 6 hpi in response to *P. pisi* and *A. euteiches*, respectively. Clusters 3 and 4 included genes mainly induced at 20 hpi in response to *A. euteiches* and *P. pisi*, respectively. Each cluster could be further divided into smaller sub-clusters containing genes with common regulatory patterns (Fig. 2, Additional file 5). Sub-clusters 1b and 2a included genes induced in response to both pathogens at 6 hpi, while sub-clusters 1a and 2b included genes specifically induced in response to *P. pisi* and *A. euteiches* at 6 hpi, respectively. Sub-clusters 3b and 4a included genes induced in response to both pathogens at 20 hpi, while sub-clusters 3a and 4b represented genes specifically induced at 20 hpi in response to *A. euteiches* and *P. pisi*, respectively (Fig. 2, Additional file 5).

The combined genes present in clusters 1 and 2 were significantly (*P* ≤ 0.005) enriched in five molecular functions and six cellular component GO categories compared with the combined genes present in clusters 3 and 4. The enriched molecular functions in clusters 1 and 2, which are mainly those induced at 6 hpi, were associated with antioxidant activity (GO:0016209), peroxidase activity (GO:0004601) and peptidase activity (GO:0008234/GO:0004197) as well as cellular component ontologies associated with cell (GO:0005623) and plant-type cell wall (GO:0009505) (Additional file 6). However, the combined genes present in clusters 3 and 4, or any combination of sub-clusters, were not enriched in any functional categories.

#### *Phytophthora pisi* and *A. euteiches* infections lead to disparate pathogen perception and signalling transcriptomic responses in pea

**Fig. 3:**
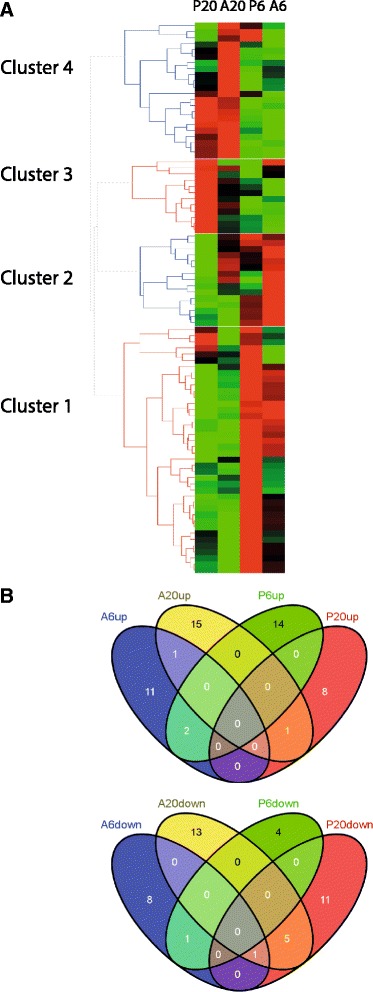
Differentially expressed pea immune signalling candidate genes. **a** Hierarchical clustering of differentially expressed genes (*P* ≤ 0.05, ≥ 1.5 fold induction or ≤ 0.67 fold repression) that corresponds to pathogen perception and signalling during early time of interaction with *A. euteiches* and *P. pisi.* The clustering is generated by HCE3.5 software with the complete linkage method and the Manhattan distance measure. Red and green represent up regulated and down regulated genes, respectively. P6 and P20 correspond to infection by *P. pisi* at 6 and 20 hpi, while A6 and A20 correspond to infection by *A. euteiches* at 6 and 20 hpi, respectively. The overlap between (**b**) up regulated and (**c**) down regulated gene sets associated with immune signalling is shown

To investigate transcriptional changes in gene classes involved in pathogen perception and signalling, genes with GO number associated with the signal transduction process (GO:0007165) were identified. In total, 89 differentially regulated genes were found among all data sets. Hierarchical clustering of genes associated with signal transduction showed four distinguished clusters, similar to those of all differentially expressed genes. The clusters represented genes uniquely induced in response to each pathogen at different time points and genes activated in common for both pathogens (Fig. 3a, Additional file 7). Cluster 1 represented a set of 40 genes that were induced at 6 hpi in response to both pathogens, but suppressed at 20 hpi. Cluster 2 represented 15 genes that were significantly induced at 6 hpi in response to *A. euteiches* and cluster 3 represented genes induced at 20 hpi in response to *P. pisi* while cluster 4 includes the genes all specifically up-regulated in response to *A. euteiches* at 20 hpi (Additional file 7). The overlap between differentially expressed genes associated with signal transduction in response to *A. euteiches* and *P. pisi* over time was determined (Fig. 3b). A majority of the genes were specifically up regulated or down regulated at each time points in response to each pathogen.

**Fig. 4:**
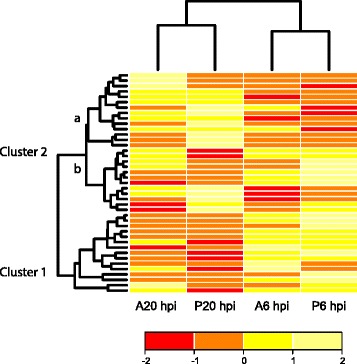
Differentially expressed pea resistance genes. Hierarchical clustering of differentially expressed pea *NB-LRR* and *RLK* genes (*P* ≤ 0.05, ≥ 1.5 fold induction or ≤ 0.67 fold repression) during early time of interaction with *A. euteiches* (A) and *P. pisi* (P)*.* The clustering is generated in R software. Yellow and red represent up regulated and down regulated genes, respectively

Among the genes associated with signal transduction, 41 were identified as putative *R*-genes and receptor-like kinases (*RLK*-genes) (Additional file 7). Hierarchical clustering of these genes revealed two clusters. Cluster 1 represented genes induced at 6 hpi while suppressed at 20 hpi. Cluster 2 included two subclasses in which subclass 2a represented genes that were suppressed at 6 hpi while induced at 20 hpi in response to both species while cluster 2b included genes that were specifically induced or suppressed at each time point responding to the pathogens (Fig. 4). Twelve and ten putative *R*- and *RLKs*-genes were down regulated at both time points upon infection with *A. euteiches* and *P. pisi*, respectively, and may thus represent potential targets of defence-suppressing pathogen effectors.

#### *Phytophthora pisi* and *A. euteiches* infections result in differential expression of defence related genes in phenylpropanoid and hormonal pathways

Downstream pea immune responses were investigated by mapping all genes to KEGG pathways and by grouping genes into two sets previously reported to be involved in plant immune responses: the phenylpropanoid pathway and biosynthesis of secondary metabolites, and hormonal signalling.

##### Phenylpropanoid pathway and biosynthesis of secondary metabolites

**Fig. 5:**
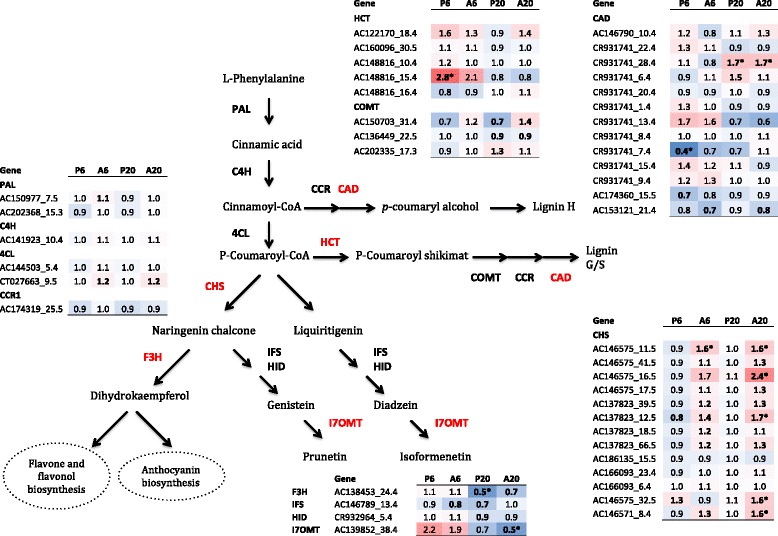
Scheme of the phenylpropanoid pathway. The expression profiles of all pea genes, which are responsive to at least one treatment (*P* ≤ 0.05), involved in the phenylpropanoid pathway during early interaction with *A. euteiches* and *P. pisi* are shown in the tables in the figure. Numbers in the coloured boxes represent the fold change (FC) in expression. P6 and A6 correspond to FC at 6 hpi while P20 and A20 correspond to FC at 20 hpi in response to *P. pisi* and *A. euteiches*, respectively, compared to mock-inoculated control samples*.* Asterisks indicate FC values that are differentially expressed (0.67 > FC > 1.5). The heat map goes from blue to red with increasing expression values. Arrows represent enzymatic reactions. The abbreviations marked in red indicate that at least one gene member of the family is differentially regulated at least in one treatment. Abbreviations: *phenylalanine ammonia-lyase* (*PAL*), *cinnamic acid 4-hydroxylase* (*C4H*), *4-coumarate-CoA ligase* (*4CL*), *cinnamoyl-CoA reductase* (*CCR*), *shikimate O-hydroxycinnamoyltransferase* (*HCT*), *caffeoyl-CoA O-methyltransferase* (*COMT*), *cinnamyl-alcohol dehydrogenase* (*CAD*), *chalcone synthase* (*CHS*), *naringin 3-dioxygenase* (*F3H*), *2-hydroxyisoflavanone synthase* (*IFS*), *2-hydroxyisoflavanone dehydratase* (*HID*) and *isoflavone O-methyltransferase* (*I7OMT*)

The gene AC148816_15.4, putatively encoding a shikimate O-hydroxycinnamoyltransferase (HCT; EC: 2.3.1.133) was induced 2.8 fold at 6 hpi in response to *P. pisi*. Five putative chalcone synthases (CHS; EC: 2.3.1.74), AC146575_11.5, AC146575_16.5, AC137823_12.5, AC146575_32.5 and AC146571_8.4) involved in the early steps of flavonoid biosynthesis, were up regulated at 20 hpi in response to *A. euteiches* (from 1.6 to 2.4 fold), but not induced in response to *P. pisi.* The putative isoflavone 7-O-methyltransferase (I7OMT; EC: 2.1.1.150) AC139852_38.4, which presumably methylates 7,4-dihydroxyiso-flavone (daidzein) and 5,7,4-trihydroxyisoflavone (genistein) to yield isoformononetin and prunetin, was suppressed 2 fold (ratio treatment/control = 0.5) at 20 hpi in response to *A. euteiches* (Fig. 5).

Genes involved in cell wall modifications were also differentially regulated. The gene CR931741_28.4, putatively encoding a cinnamyl-alcohol dehydrogenase (CAD; EC: 1.1.1.195) that is responsible for the last enzymatic step in the monolignol biosynthesis, was induced 1.7 fold at 20 hpi in response to both pathogens, while another member of this gene family, CR931741_7.4 was suppressed 2.6 fold at 6 hpi in response to *P. pisi* (Fig. 5). Furthermore, two putative callose synthases (GSL; EC: 2.4.1.34) AC155803_41.5 and AC137603_39.4, were up regulated 1.6 fold at 6 hpi in response to *P. pisi* and *A. euteiches*, respectively. However, at 20 hpi *GSL* AC155803_41.5 showed suppression by 2 fold in response to both pathogens (Table 2). Several putative pectin esterase (EC: 3.1.1.11) genes were differentially expressed; two different genes, AC174141_27.4 and CT009653_39.4, were induced 1.5 and 1.7 fold against *A. euteiches* at 6 and 20 hpi, respectively, while three other genes were suppressed upon infection by *P. pisi* (AC150204_16.5 at 6 hpi, AC153005_9.5 and CT009653_39.4 at 20 hpi). The gene AC173289_18.5, encoding a putative pectin esterase inhibitor enzyme (EC: 3.1.1.11) predicted to prevent or reduce the activity of pectin esterases, was induced at 20 hpi 1.6 and 1.7 fold during interaction with *A. euteiches* and *P. pisi,* respectively (Table 2).

**Table 2:** Expression of pea genes involved in cell wall modifications

Description	Gene^a^	P6hpi	A6hpi	P20hpi	A20hpi
Callose synthase	AC137603_39.4	1.0	**1.6** ^**b**^	1.2	0.8
	AC155803_42.5	1.0	**1.4**	0.8	1.0
	AC155803_41.5	**1.6** ^**b**^	0.9	0.5	**0.5** ^**b**^
	AC155803_43.5	**1.2**	0.9	1.0	0.9
	AC122723_35.5	1.1	1.1	1.1	0.9
	CU012050_22.4	1.0	0.9	1.0	1.2
	AC202574_37.3	1.1	1.1	1.0	**0.8**
Pectinesterase	AC148775_45.5	0.9	**0.8**	1.0	1.0
	AC150204_16.5	**0.6** ^**b**^	0.8	0.9	1.0
	AC153005_36.5	1.0	**1.2**	0.9	1.1
	AC153005_9.5	0.9	0.7	**0.3** ^**b**^	0.7
	AC174141_27.4	1.3	**1.5** ^**b**^	1.1	1.1
	AC202348_3.4	1.0	1.1	1.0	**1.3**
	CT009653_39.4	1.0	1.0	1.3	**1.7** ^**b**^
	AC152919_7.5	1.0	0.8	**0.6** ^**b**^	**0.7**
Pectinesterase inhibitor	AC122165_33.4	1.2	1.0	**1.6** ^**b**^	1.4
	AC160097_13.5	0.9	0.9	1.0	1.0
	AC160097_28.5	0.9	0.9	1.0	**1.4**
	AC165218_17.4	**1.3**	1.3	1.0	1.1
	AC173289_18.5	1.1	1.3	1.7	**1.6** ^**b**^

^a^Refers to the gene accession numbers in *M. truncatula* A17 genome version 2 (Mt2.0)

Numbers indicate fold change (FC) ratios of expression levels of genes involved in hormonal signalling at 6 hpi and 20 hpi in response to *A. euteiches* and *P. pisi* compared with mock inoculation. A6hpi and P20hpi correspond to the expression values in response to *A. euteiches* at 6 hpi and 20 hpi, respectively, while P6hpi and P20hpi correspond to the expression values in response to *P. pisi* at 6 hpi and 20 hpi, respectively. Bold values indicate if the treatment FC is significantly (*P* ≤ 0.05) different from the control. ^**b**^Indicate if the FC expression levels are differentially expressed (*P* ≤ 0.05, ≥ 1.5 fold induction or ≤ 0.67 fold repression)

##### Hormonal signalling

At 6 hpi the gene AC155803_43.5, putatively encoding a lipoxygenase (LOX; EC: 1.13.11.12) that is involved in the biosynthesis of JA, was suppressed by 1.6 fold in response to both pathogens while two other members of this gene family, AC146571_7.4 and AY515253_32.4, were induced at 20 hpi in response to *P. pisi* and *A. euteiches* by 2.3 and 1.5 fold, respectively. Two (AC169513_37.4 and AC125389_65.5) and three (AC146817_41.4, AC174337_15.4 and AC202309_24.3) putative *ACO* genes, involved in the final step of ET production, were up regulated upon infection with *P. pisi* at 6 and 20 hpi, respectively (from 1.5 to 2.9 fold). In contrast, two other putative *ACO* genes, AC169513_37.4 and AC169513_37.4, showed 1.8 fold inductions in response to *A. euteiches* at 6 hpi and 20 hpi, respectively. The AC145767_15.4 gene that is a putative *ethylene-responsive transcription factor* (*ERF*) gene, essential for ET biosynthesis [26], showed 1.6 fold induction at 20 hpi against *A. euteiches* only. Three putative auxin-induced SAUR family member genes (CU326390_14.3, AC148242_50.4 and AC146705_13.5), known to be rapidly and transiently up regulated in response to auxin [27, 28], were induced against *A. euteiches* at 20 hpi (by 1.9 fold) but constitutively expressed against *P. pisi* (Table 3).

**Table 3:** Expression of pea genes involved in hormonal signalling pathways

Description	Gene^a^	P6hpi	A6hpi	P20hpi	A20hpi
Lipoxygenase (LOX)	AY515253_32.4	1.0	0.8	1.2	**1.5** ^**b**^
	AC146571_7.4	0.7	**0.7**	**2.3** ^**b**^	1.3
	AC140032_2.4	0.8	0.8	**1.5** ^**b**^	1.3
	AC149580_13.5	**0.6** ^**b**^	**0.6** ^**b**^	1.0	0.7
	AC149638_35.4	0.9	1.1	0.8	**0.7**
	AC149580_9.5	0.9	0.9	1.0	**0.8**
	AC149580_19.5	0.9	1.0	**0.8**	**0.9**
	AC140032_6.4	0.9	1.0	0.9	**0.8**
	AC140032_7.4	1.0	1.1	1.0	1.0
Jasmonate O-methyltransferase	AC152936_2.5	1.6	0.9	1.3	**1.7** ^**b**^
	CU024875_36.4	1.1	1.1	**0.7**	**0.8**
Aminocyclopropane-carboxylate oxidase (ACO)	AC146817_44.4	1.1	**1.4**	1.2	1.2
	AC174337_3.4	1.0	**1.3**	0.8	1.3
	AC169513_37.4	**2.0** ^**b**^	**1.8** ^**b**^	0.6	0.9
	AC125389_65.5	**1.6** ^**b**^	1.3	1.1	0.9
	AC119419_24.4	**1.4**	1.0	0.8	0.8
	AC158372_38.4	1.5	1.4	**0.6** ^**b**^	0.7
	AC146817_41.4	0.8	0.5	**2.9** ^**b**^	2.1
	AC174337_15.4	1.2	0.9	**2.0** ^**b**^	1.4
	AC202309_24.3	1.3	1.1	**1.5** ^**b**^	**0.7**
	AC124966_44.4	1.3	1.2	0.9	**1.8** ^**b**^
	CT025839_48.5	1.0	**0.5** ^**b**^	1.2	1.4
	CU013517_26.4	1.0	0.9	**1.3**	1.0
	AC197464_13.4	1.0	1.0	**0.9**	**0.8**
	AC158372_65.4	1.0	1.1	0.7	**0.6** ^**b**^
	AC169513_36.4	0.9	1.0	0.8	**0.7**
	AC158372_67.4	0.7	0.8	0.7	**0.7**
	AC158372_42.4	1.0	1.0	0.9	**0.7**
	AC169513_23.4	0.8	**0.7**	0.9	1.0
	AC169513_26.4	0.9	**0.8**	1.0	1.1
Ethylene-responsive transcription factor (ERF)	AC145767_15.4	1.0	1.0	1.3	**1.6**
Ethylene insensitive 3 (EIN3)	AC196764_17.3	0.9	0.9	1.0	**1.3**
Auxin-induced protein (SAUR)	CU326390_14.3	1.1	1.1	1.3	**1.9** ^**b**^
	AC149578_14.4	1.0	1.0	**1.3**	**1.3**
	AC148242_22.4	1.1	1.0	**1.4**	1.2
	AC148242_37.4	1.0	1.0	1.2	**1.3**
	AC148242_50.4	1.1	1.3	1.2	**1.7** ^**b**^
	AC148242_51.4	1.0	1.0	1.1	**1.4**
	AC146705_44.5	1.0	1.1	**1.2**	**1.2**
	AC146705_10.5	1.1	1.0	1.2	**1.3**
	AC146705_14.5	1.1	1.1	**1.2**	**1.1**
	AC146705_13.5	0.9	1.0	1.2	**1.7** ^**b**^
	AC146705_14.5	1.1	1.1	**1.2**	**1.1**
	CU024876_8.4	0.9	1.1	**1.3**	1.1
	CU024876_38.4	1.1	1.1	**1.3**	**1.2**
	AC146705_4.5	**1.4**	1.2	0.8	0.8
Auxin transporter-like protein 2-like	CT030165_15.5	1.0	1.0	0.9	**0.8**
Auxin-responsive protein	CU459036_7.3	1.0	1.0	1.0	**0.9**
	AC152423_24.4	1.0	0.9	**1.3**	1.1
Cullin-like protein1	AC150246_30.4	1.5	0.9	0.7	**0.3**
	CR931807_17.5	**4.9** ^**b**^	1.3	1.4	0.6
	CR956619_45.5	**2.2** ^**b**^	1.3	0.7	0.8
	CT573078_55.5	**2.3** ^**b**^	1.7	0.3	0.5
	AC121235_22.5	1.7	**2.1** ^**b**^	1.2	1.3
Pathogenesis-related protein 1A	AC150778_28.5	1.1	1.3	1.2	**2.6** ^**b**^
Abscisic acid receptor PYL4-like	CT967319_16.4	1.0	1.0	1.0	**1.1**
Abscisic acid-insensitive 5-like protein 2-like	AC146910_18.5	**1.2**	1.2	0.9	1.1
Gibberellin receptor GID1c-like	AC188382_8.4	1.0	1.0	0.9	**0.9**
F-box family protein	AC146792_3.5	0.9	1.0	1.0	**0.8**
Transcription factor HBP-1b -like	AC157891_47.4	1.0	1.0	**0.9**	0.9
Two-component response regulator ARR9-like	AC153125_5.5	0.7	**0.4** ^**b**^	**0.5** ^**b**^	**0.5** ^**b**^
TGACG-sequence-specific DNA-binding protein	AC202316_14.3	0.9	0.9	**0.8**	0.9
BTB/POZ ankyrin repeat protein	AC147961_14.5	0.9	1.0	**0.9**	0.9
Protein phosphatase 2C 37-like	CR954191_11.4	0.9	**0.9**	0.9	1.0
Acyl-CoA oxidase	CT573502_1.5	1.1	**1.1**	1.1	**1.2**

^a^Refers to the gene accession numbers in *M. truncatula* A17 genome version 2 (Mt2.0)

Numbers indicate fold change (FC) ratios of expression levels of genes involved in hormonal signalling at 6 hpi and 20 hpi in response to *A. euteiches* and *P. pisi* compared with mock inoculation. A6hpi and A20hpi correspond to the expression values in response to *A. euteiches* at 6 hpi and 20 hpi, respectively, while P6hpi and P20hpi correspond to the expression values in response to *P. pisi* at 6 hpi and 20 hpi, respectively. Bold values indicate if the treatment FC is significantly (*P* ≤ 0.05) different from the control. ^**b**^Indicate if the FC expression levels are differentially expressed (*P* ≤ 0.05, ≥ 1.5 fold induction or ≤ 0.67 fold repression)

### Confirmation and investigation of differential gene expression by quantitative PCR

**Fig. 6:**
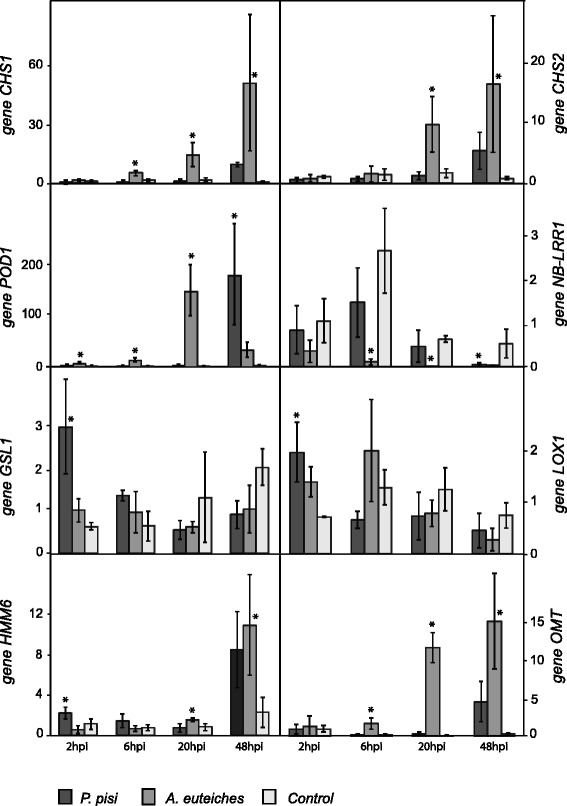
Expression profiles of selected pea genes during interaction with *A. euteiches* and *P. pisi*. Relative expression levels were normalized by *β*-tubulin (*TUB*) expression, and presented in relation to control pea plants at 2 hpi (= expression level 1) using the 2^-∆∆Ct^ formula. Error bars represent standard deviation based on at least 4 biological replicates. Asterisks indicate statistically significant (*P* ≤ 0.05) differences between infection treatments and control plants within time points according to the Fisher test. Abbreviations: *chalcone synthesis* (*CHS1*, *CHS2*), *peroxidase* (*POD1*), *nucleotide binding - leucine rich repeat resistance* (*NB-LRR1*), *callose synthase* (*GSL1*), *lipoxygenase* (*LOX1*), *6a-hydroxymaackiain methyltransferase* (*HMM6*) and O*-methyltransferase* (*OMT*)

RT-qPCR was used to validate the microarray data and to assess the expression levels of seven selected candidate genes in pea during interaction with *P. pisi* and *A. euteiches* at 2 hpi, 6 hpi, 20 hpi and 48 hpi. The genes were chosen to include genes specifically induced, suppressed and non-regulated in response to *A. euteiches*, and to cover several different functional categories. The expression patterns observed by RT-qPCR were in agreement with those obtained by microarray at 6 hpi and 20 hpi (Fig. 6). The only exception was that the pea *OMT* gene, which showed similarity (E-value 0.0 and 72 % identity) to the *M. truncatula I7OMT* involved in methylation of daidzein and genistein, was down regulated at 20 hpi in response to *A. euteiches* according to the microarray experiment but up regulated at this time according to RT-qPCR (*P* < 0.001). The expression pattern of the *HMM6* gene, putatively encoding a 6a-hydroxymaackiain methyltransferase enzyme that catalyses the last step of the synthesis of the main pea phytoalexin pisatin, was investigated by RT-qPCR although no data was available for this gene in the microarray experiment. The result showed up-regulation of this gene against *A. euteiches* at 20 hpi compared to the control, followed by 10 fold induction at 48 hpi compared to 2 hpi (*P* ≤ 0.029).

In response to *A. euteiches*, the putative chalcone synthase genes *CHS1* and *CHS2* were induced 10 fold compared to the control at 20 hpi (*P* ≤ 0.006), while they were constitutively expressed during *P. pisi* infection. The *GSL1* gene, putatively encoding a callose synthase, was induced at 2 hpi specifically in interaction with *P. pisi* (*P* < 0.007). The *POD1* gene putatively encoding a peroxidase was specifically induced during the 2-20 hpi interaction with *A. euteiches* (*P* ≤ 0.007) while only induced at 48 hpi in response to *P. pisi* (*P* = 0.009). The *NB-LRR1* gene, putatively encoding a nucleotide binding-site leucine-rich repeat protein, was specifically suppressed by 6 hpi after *A. euteiches* infection and later during that interaction (*P* ≤ 0.012). The expression of the *LOX1* gene, putatively encoding a linoleate 13S-lipoxygenase, was up regulated early after infection with *P. pisi* (at 2 hpi, *P* = 0.010) while constitutively expressed later during infection with both pathogens.

## Discussion

Our previous characterization of the infection process of *P. pisi* on pea roots suggests that the infection process turns necrotrophic by 27 hpi [29]. However, haustorium formation in the prospective biotrophic phase is not reported in either *A. euteiches* or *P. pisi*. Based on the significant gene expression induction of defence marker genes *Pi49, ABA17*, *ACO* and *chit4* [30, 31] at 20 and 48 hpi*,* and the evaluation of the infection process in a previous study [29], pea roots at 6 and 20 hpi were sampled for the transcriptomic analysis to represent early infection.

A number of reference genomes for legumes including *M. truncatula, Glycine max* and *Phaseolus vulgaris* are completed [32–34], but the genome of pea has not yet been sequenced and this limits the usefulness of deep sequencing approaches for transcriptomic investigations. An approach to study the transcriptional responses in species lacking genome sequence information is heterologous probing on microarrays based on closely related species, which is successfully used for pea by hybridization on a *M. truncatula* microarray [14].

Sources of bias and errors when using an heterologous probing approach to analyse gene expression patterns are i) the efficiency of the hybridizations and ii) variations in gene family structure and gene sequence between species [35]. Therefore, it is important to validate results obtained with microarray, with an alternative approach such as RT-qPCR. The expression analyses of the pea *OMT* and *M. truncatula I7OMT* gene illustrates these problems; Our microarray experiment showed that a gene similar to *M. truncatula I7OMT* was suppressed in response to *A. euteiches* at 20 hpi but the results obtained by RT-qPCR for a predicted pea *O*-methyltransferase (*OMT*) transcript with high similarity to *M. truncatula I7OMT* (75 % amino acid identity), obtained from a pea RNA-seq assembly [8], showed induction of this gene at 20 hpi. Thus, the actual variation between the two *I7OMT*/*OMT* sequences, or potential variations in the *OMT* gene family structure between the two species, interfered with the analysis. However the results of RT-qPCR for other target genes showed common expression pattern as in microarray, confirming the microarray analysis and indicating that the use of the *M. truncatula* microarray for studying the pea transcriptome is a reliable tool, as it was reported previously [14].

In the current work, we study compatible interactions between pea and the two pathogens *A. euteiches* and *P. pisi*, which results in disease. We hypothesise that in these compatible interactions the transcriptomic responses in pea are linked with immunity, and thus represent a failed defence response. However, there are examples of susceptibility genes in pea. The *PsMLO1* gene, coding for a plant specific membrane protein with as yet unknown function, is an example of a susceptibility gene in pea against powdery mildew caused by *Erysiphe pisi* [36]. No susceptibility genes associated with *A. euteiches* or *P. pisi* are as yet identified in pea.

Comparison between time points for each pathogen and interspecies infection revealed distinct sets of differentially regulated genes in response to *A. euteiches* and *P. pisi* and at each time point. This indicates that the different pathogenicity mechanisms of *A. euteiches* or *P. pisi* lead to disparate transcriptional changes in pea. This interpretation is strengthened by the expression patterns of genes involved in pathogen perception and signalling where different sets of genes are specifically differentially regulated in response to each pathogen, indicating that different signalling molecules in pea are triggered by these two oomycetes.

Furthermore, the induction and suppression of genes associated with signal transduction pathways at 6 hpi in response to both pathogens suggest that immunity responses associated with PTI or ETI occurs early in infection. Interestingly, many genes encoding NB-LRRs are suppressed early after infection, indicating that these oomycetes secrete and deliver their effectors into the pea root cells early during infection to suppress immune signalling leading to ETS. This idea is in line with the transcriptomic analysis of *P. capsici*-tomato interaction, where a subset of pathogen effectors and host receptor genes were induced and repressed, respectively, during biotrophy [37]. Furthermore, the data indicates that some NB-LRR and LRR-RLK encoding genes in pea are triggered commonly against both pathogens while some are specifically activated at each time point responding to each of these oomycetes.

Transcriptional changes suggesting increased cell wall reinforcement are observed in response to both pathogens. Cell wall reinforcement contributes to the development of physical barriers through deposition of cell wall appositions at sites of pathogen detection as a common component of the PTI response [19, 38]. *GSL* genes of higher plants encode essential proteins for callose formation [39]. Therefore, induction of pea putative *GSL* genes early after infection against these oomycetes suggests the formation of callose at the infection site as a part of the defence mechanism. This is in agreement with a study where callose deposition was shown as a defence mechanism to restrict *P. capsici* growth at early time in an incompatible interaction with *Arabidopsis thaliana* [40]. Induction of a putative *CAD* gene, involved in lignification, suggests that lignin deposition in the cell walls is part of the defence mechanism against both *A. euteiches* and *P. pisi*. This result is consistent with a study where lignin deposition in cell walls is shown to be a striking feature of *M. truncatula* partial resistance against *A. euteiches* [41].

Based on the expression pattern of the putative *LOX* genes in response to *P. pisi* and *A. euteiches*, it appears that JA biosynthesis is down regulated at 6 hpi and activated at 20 hpi. A crucial role of LOX compounds in resistance of tobacco in an incompatible interaction with *P. parasitica* was shown [42]. Furthermore, an increase in susceptibility of JA-deficient mutant tomato (*Lycopersicon esculentum*) plants to *P. infestans* has been reported [43]. Therefore, considering the role of JA as an important mediator in defence signalling against these oomycetes, we hypothesize that *P. pisi* and *A. euteiches* suppress JA biosynthesis in pea during the early phases of susceptible interactions for favouring the infection. Induction of JA pathway at 20 hpi in pea is in agreement with a recent study on soybean - *P. sojae* interaction where the JA pathway was up regulated at 24 hpi in the susceptible soybean lines [44]. On the other hand, induction of *ACO* genes that is positively correlated with ethylene production rates [45] suggests up regulation of ET biosynthesis pathway in pea upon infection with both pathogens. While ET and JA are often regarded to be part of the same signalling module, a negative regulatory relationship between components of these two pathways is reported [19]. Furthermore, non-synergetic regulation of these two pathways is reported in compatible and incompatible soybean - *P. sojae* interactions at 24 hpi [44]. Activation of the ET pathway in response to *A. euteiches* is supported by induction of *ERF*, which is the transcription factor essential in ET signalling [46].

In contrast to the similarities in the regulation of JA and ET biosynthesis genes in pea during interactions with *P. pisi* and *A. euteiches*, induction of genes encoding auxin-induced SAUR family proteins appears to be specific to *A. euteiches*. The induction of SAUR family proteins may indicate an accumulation of auxin in roots during *A. euteiches* infection. Auxin signalling is reported to be important in *A. thaliana* resistance against oomycetes such as *Pythium irregulare* and *Hyaloperonospora arabidopsidis* [47, 48] and repression of this pathway enhances the susceptibility of *A. thaliana* to *P. cinnamomi* [49]. Recently, the polar auxin transport in roots was reported to be targeted by an RXLR effector of *P. parasitica*, the Penetration-Specific Effector 1 (PSE1), in compatible interactions with *A. thaliana* roots [50], leading to modulation of the auxin content (possibly by lowering the auxin concentrations) locally at the root apex to favour infection. A possible interpretation of our data is that *P. pisi*, but not *A. euteiches*, possess effectors that target and suppress auxin accumulation in pea roots, thereby favouring infection.

Induction of *CHS* genes putatively encoding chalcone synthases also appears to be specific to infection by *A. euteiches.* Naringenin chalcone that is the product of the CHS reaction, is a substrate for the production of a wide range of secondary metabolites, including flavones, isoflavonoid phytoalexins, and anthocyanins. Legumes utilize flavonoid compounds, notably isoflavones and isoflavanones in defence against pathogens and as signalling molecules [22]. Induction of genes in the phenylpropanoid pathway, such as *phenylalanine ammonia-lyase* (*PAL*), *CHS* and isoflavone synthase, was shown to be rapid and strong in compatible interaction in soybean against *P. sojae* [51]. Furthermore, induction of genes involved in this pathway was reported in *M. truncatula* in response to pathogen infection such as *Erysiphe pisi* and *Colletotrichum trifolii* [52–54]. Taken together, the induction of *CHS* genes during infection by *A. euteiches* suggests that biosynthesis of secondary metabolites e.g. isoflavonoid phytoalexins, is a part of plant immunity response to this pathogen.

Local resistance of pea root tips against *A. euteiches* is previously reported to be associated with an increase in pisatin production in the border cells [4], and silencing of *HMM6* results in reduced pisatin production [55]. Induction of pea *HMM6* in response to *A. euteiches* suggests that pisatin synthesis might be part of the defence response against this pathogen.

## Conclusions

**Fig. 7:**
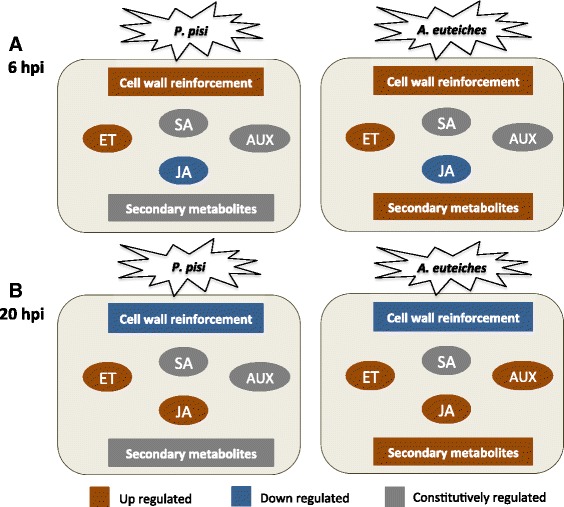
Differentially regulated defense sectors in pea. Summary of defense-related transcriptional differences in pea during compatible interactions with *Aphanomyces euteiches* and *Phytophthora pisi* at 6 hpi (**a**) and 20 hpi (**b**). Abbreviations: Ethylene pathway (ET), Salicylic acid pathway (SA), Auxin pathway (AUX), Jasmonic acid pathway (JA)

Our results show that different gene sets are triggered in pea by *A. euteiches* and *P. pisi*, leading to distinct and common transcriptional responses during the early phase of susceptible interaction with these two distantly related oomycetes. Cell wall reinforcement and modulation of JA and ET pathways are similar in response to both pathogens, while induction of the auxin pathway and chalcone synthesis is specific response to *A. euteiches* (Fig. 7). Taken together, this knowledge will lead to a better understanding of the early defence response in pea against these important pathogens. Future advances in our understanding of oomycete infection mechanisms will explain more of the distinct patterns we observe.

## Methods

### Plant material and inoculation

Pea seeds, cv. “Finulf”, were surface sterilized in sodium hypochlorite (10 % *v/v*) for 3 min, and washed in water. The seeds were then germinated in moist autoclaved paper towels by incubation in darkness at 25 °C for four days. Strain 97603 of *P. pisi* and strain 11 k3 of *A. euteiches* were grown on dilute vegetable Granini Juice agar (4 % filtered juice and 2 % Bacto Agar) at 25 °C. Zoospores of these two species were produced as described previously [29]. The concentration of zoospores was further determined with a haemocytometer and adjusted to 10^5^ zoospores per ml. Germinated pea seedlings with approximately 4 cm roots were selected and the roots were incubated in the zoospore suspension of each species for 30 min in a previously described infection system [29], followed by incubation in a growth chamber with 16 h light at 22 °C and high humidity. Roots were harvested at 0, 30 min, 1, 2, 6, 20 and 48 hpi. At each time point, mock-inoculated control plants grown under the same conditions were harvested. Each treatment was performed in three replicates, each consisting of 10 pea roots. For nucleic acid extractions, the distal ends of the pea roots were cut about 1 cm from the tips, ground in liquid N_2_ and stored at –80 °C.

### Nucleic acid isolation and DNase I treatment

Total RNA was extracted using a phenol-chloroform protocol as described by Dubey et al. [56], followed by NaOAc/ethanol purification and proceeded to the RNeasy Plant Mini Kit (Qiagen, Hilden, Germany) according to the manufacturer’s instructions. Traces of DNA were removed by DNase I treatment (Fermentas). RNA concentration was determined spectrophotometrically using Nano-Drop (Thermo Scientific), and RNA quality was assessed after electrophoresis on an Agilent Bioanalyzer using the RNA 6000 nano kit (Agilent Technologies, Santa Clara, CA) according to the manufacturer’s instructions. DNase I treated RNA was further diluted to 1-2 μg/μl for microarray analysis.

### Microarray experiment and analysis

Samples taken at 6 and 20 hpi were selected for the microarray experiment. The microarray hybridizations were performed at Swegene center for integrative biology at Lund University, Sweden. 200 ng of DNAse I treated samples were used for cDNA synthesis and labeling by Biotin Allonamide Triphosphate at 37 °C. The Medicago MedGene-1-0-st array, designed based on the *M. truncatula* A17 genome version 2 (Mt2.0), was used for this experiment. The experiment included three biological and two technical replicates incorporating one dye swap.

Basic Affymetrix chip and Experimental Quality Analyses were performed using the Expression Console Software V1.1.2. Probe summarization and data normalization method, Robust Multi-array Analysis (RMA) was done as described by Irizarry et al. [57]. Signals were log_2_ transformed. To identify differentially expressed genes in inoculated samples at each time point compared to control samples, the Limma model [25] in the R software was used [58]. Genes that were statistically differently regulated at each time point compared to the corresponding control samples (*P* ≤ 0.05), were considered as responsive genes to that time point. Among these genes, those with log_2_ expression ratio treatment/control ≥ 0.584 (>1.5 fold induction or ≤ 0.67 repression) were regarded as differentially expressed genes. Venn diagrams of differentially expressed gene sets were generated using Venny [59]. Hierarchical clustering of all genes that were differentially expressed at least in one condition was performed using the HCE3.5 software [60] with the complete linkage method and the Manhattan measure. Hierarchical clustering of *R* genes was generated in R software. Blast2GO [61] analysis was performed to provide Gene Ontology annotation according to BlastP [62] hits against the NCBI with an e-value threshold of 1e-6. Functional category assignment for differentially expressed genes was conducted using the WEGO online server [63], classifying according to GO terms within molecular functions, biological processes and cellular components. KEGG Automatic Annotation Server (KAAS) Ver. 1.69x [64] was used to annotate protein sequences using orthologs of plant enzymes (*A. thaliana* and *G. max*) to obtain KEGG Orthology (KO) and Enzyme Commission (EC) numbers. The enzyme EC codes were mapped to the KEGG database [65] using the KEGG mapper-reconstruct pathways tool (Ver. 2.0) [66]. Domain structure and family of possible resistance genes were investigated using the InterPro online server [67]. Microarray data have been deposited in ArrayExpress (http://www.ebi.ac.uk/arrayexpress/) under accession number E-MTAB-3748.

### Assessment of gene expression using RT-qPCR

For validation of microarray data, eight *P. sativum* genes with similarity (≥70 % amino acid identity) to *M. truncatula* genes previously reported to be involved in defence were retrieved from NCBI or pea transcriptome data [8]. Primers used for RT-qPCR were designed towards sequences of pea genes to amplify amplicons ranging from 80 to 200 bp using Primer Select software (Dnastar, Madison, WI) (Additional file 8).

Reverse transcription of 1 μg of the same DNase I treated RNA used for microarray analysis was carried out in a 20 μl reaction volume using the iScript cDNA Synthesis Kit (Bio-Rad, Hercules, CA) according to the manufacturer’s instructions. The synthesized cDNA was diluted 10× into a final volume of 200 μl. Transcript levels were quantified using an iQ5 qPCR System (Bio-Rad, Hercules, CA). Each 20 μl reaction contained 5 μl of diluted cDNA, 150 nM of each primer and 10 μl SsoFast EvaGreen Supermix (Bio-Rad, Hercules, CA). The following PCR protocol was used: 98 °C for 2 min, 40 cycles of 98 °C for 5 s, 60 °C for 10 s and 72 ° C for 10 s. Amplification of a single product was confirmed by melt curve analysis, while primer amplification efficiency was deducted from amplification of standard curves using dilution series of genomic DNA.

Relative expression values for *P. sativum* target genes were calculated from the Ct values according to the 2^-∆∆Ct^ method [68], and normalised by the elongation factor alfa (*EFA*) [29] or β-tubulin (*TUB*) [69] reference genes. Transcript levels were determined in 4 biological replicates, each based on 2 technical replicates. Analysis of variance (ANOVA) was conducted using a General Linear Model implemented in SPSS ver. 21 (IBM, Armonk, NY) or Statistica ver. 12 (StatSoft Inc., Tulsa, OK). Pairwise comparisons were made using Fisher’s method at the 95 % significance level.

### Availability of supporting data

The data supporting the results of this article are included within the article and its additional files.

## Additional files

Additional file 1: Figure S1. Expression profiles of selected marker pea genes during interaction with *A. euteiches* and *P. pisi.* Expression patterns of four pea genes in response to both pathogens were investigated by RT-qPCR. Relative expression levels were normalized by elongation factor alfa (*EFA*) expression, and presented in relation to *P. pisi*-infected plants at 1 hpi (= expression level 1) using the 2^-∆∆Ct^ formula. Error bars represent standard deviation based on three biological replicates. Asterisks indicate statistically significant (*P* ≤ 0.05) differences between infection treatments and control plants at 48 hpi according to the Fisher test. (PDF 427 kb) Additional file 2: Table S1. List of all pea responsive genes to infection with *A. euteiches.* Microsoft Excel spreadsheet containing complete list of pea responsive genes to *A. euteiches* infection at 6 hpi (A6UP, A6DOWN) and 20 hpi (A20UP, A20DOWN). All genes have *P* ≤ 0.05 on log_2_ of fold change (FC) expression levels compared to the control samples. Each gene is presented by the *M. truncatula* gene ID (column A), annotation (column B), minimum E-value (the lowest E-value of the ten best hits) (column C), mean similarity (the average similarity value of the ten best hits) (column D) and enzyme code (column E) provided by Blast2GO. Ratios of log_2_ on the FC between each treatment and the corresponding control samples are presented in column F. Differentially expressed genes (*P* ≤ 0.05, ≥ 1.5 fold induction or ≤ 0.67 fold repression) are marked with light blue colour. (XLSX 465 kb) Additional file 3: Table S2. List of all pea responsive genes to infection with *P. pisi*. Microsoft Excel spreadsheet containing complete list of list of pea responsive genes to *P. pisi* infection at 6 hpi (P6UP, P6DOWN) and 20 hpi (P20UP, P20DOWN). All the genes have *P* ≤ 0.05 on log_2_ of fold change (FC) expression levels to the control samples. Each gene is presented by the *M. truncatula* gene ID (column A), annotation (column B), minimum E-value (the lowest E-value of the ten best hits) (column C), mean similarity (the average similarity value of the ten best hits) (column D) and enzyme code (column E) provided by Blast2GO. Ratios of log_2_ on the FC between each treatment and the corresponded control samples are presented in column F. Differentially expressed genes (*P* ≤ 0.05, ≥ 1.5 fold induction or ≤ 0.67 fold repression) are marked with light blue colour. (XLSX 329 kb) Additional file 4: Figure S2. GO classification of differentially expressed pea genes in response to *A. euteiches* and *P. pisi.* Up regulated and down regulated genes (*P* ≤ 0.05, ≥ 1.5 fold induction or ≤ 0.67 fold repression) in response to *A. euteiches* at 6 hpi (A) and 20 hpi (B), to *P. pisi* at 6 hpi (C) and 20 hpi (D). The plots were generated using WEGO. (PDF 2895 kb) Additional file 5: Table S3. List of differentially expressed pea genes during interaction with *A. euteiches* and *P. pisi.* Microsoft Excel spreadsheet containing *M. truncatula* gene IDs (column A), annotation (column B), minimum E-value (the lowest E-value of the ten best hits, column C), mean similarity (the average similarity value of the ten best hits, column E) and enzyme code (column E) provided by Blast2GO. Ratio of the log_2_ expression between each treatment and the corresponded control samples is presented in column F to I. Genes are clustered based on their expression patterns as shown in Fig. 2. Sheets 2 and 3 represent the genes in sub-clusters 1b and 2a, respectively, which are mainly induced in response to both pathogens at 6 hpi. Sheets 1 and 4 represent the genes in sub-clusters 1a and 2b that are specifically induced in response to *P. pisi* and *A. euteiches* at 6 hpi, respectively. Sheets 6 and 7 represent the genes in sub-clusters 3b and 4a, respectively, which are induced in response to both pathogens at 20 hpi. Sheets 5 and 8 represent the genes in sub-clusters 3a and 4b that are specifically induced at 20 hpi in response to *A. euteiches* and *P. pisi*, respectively. (XLSX 280 kb) Additional file 6: Figure S3. Gene ontologies (GO) enriched in the early defence transcriptional response in pea. Percentage of genes with significantly enriched (*P* < 0.005) GO terms that were identified in cluster 1 and 2 (representing genes induced at 6 hpi in response to both pathogens), compared with the background (genes induced at 20 hpi, present in cluster 3 and 4)*. (PDF 347 kb)*
Additional file 7: Table S4. List of differentially expressed pea genes involved in signalling during interaction with *A. euteiches* and *P. pisi.* Microsoft Excel spreadsheet containing *M. truncatula* gene IDs (column A), annotation (column B), minimum E-value (the lowest E-value of the ten best hits, column C), mean similarity (the average similarity value of the ten best hits, column E) and enzyme code (column E) provided by Blast2GO. Ratio of the log_2_ expression between each treatment and the corresponded control samples is presented in column F to I. Genes are clustered based on their expression patterns as shown in Fig. 3a. Sheet 5 represents all the possible resistance genes involved in signalling pathways with the gene ID (column A), heat map of ratio of the log_2_ expression patterns (column B to E), annotation (column F) and domain structure and family generated by InterPro (column G). (XLSX 71 kb) Additional file 8: Table S5. Primers used in this study for RT-qPCR analysis. (DOCX 20 kb)

## Abbreviations

ABA: Abscisic acid
ACO: 1-aminocyclopropane-1-carboxylate oxidase
ANOVA: Analysis of variance
CAD: Cinnamyl-alcohol dehydrogenase
Chit: Chitinase
CHS: Chalcone synthases
EC: Enzyme Commission
EFA: Elongation factor alfa
ERF: Ethylene-responsive transcription factor
ET: Ethylene
ETI: Effector-triggered immunity
ETS: Effector-triggered susceptibility
GO: Gene Ontology
GSL: Callose synthases
HCT: Shikimate O-hydroxycinnamoyltransferase
HMM6: 6a-hydroxymaackiain methyltransferase
I7OMT: Isoflavone 7-O-methyltransferase
JA: Jasmonic acid
KEGG: Kyoto Encyclopedia of Genes and Genomes database
KO: KEGG Orthology
LOX: Lipoxygenase
LRR: Leucine-rich repeat protein
MAMPs: Microbe-associated molecular patterns
NB: Nucleotide binding domain
OMT: O-methyltransferase
PAL: Phenylalanine ammonia-lyase
PAMPs: Pathogen-associated molecular patterns
POD: Peroxidase
PTI: Pattern-triggered immunity
RLK: Receptor-like kinases genes
SA: Salicylic acid
TUB: β-tubulin
