# Supplementary figures and images for: Deciphering common and specific transcriptional immune responses in pea towards the oomycete pathogens Aphanomyces euteiches and Phytophthora pisi
Source: BMC Genomics. 2015 Aug 21;16(1):627. doi: 10.1186/s12864-015-1829-1 (PMC4546216; doi:10.1186/s12864-015-1829-1)

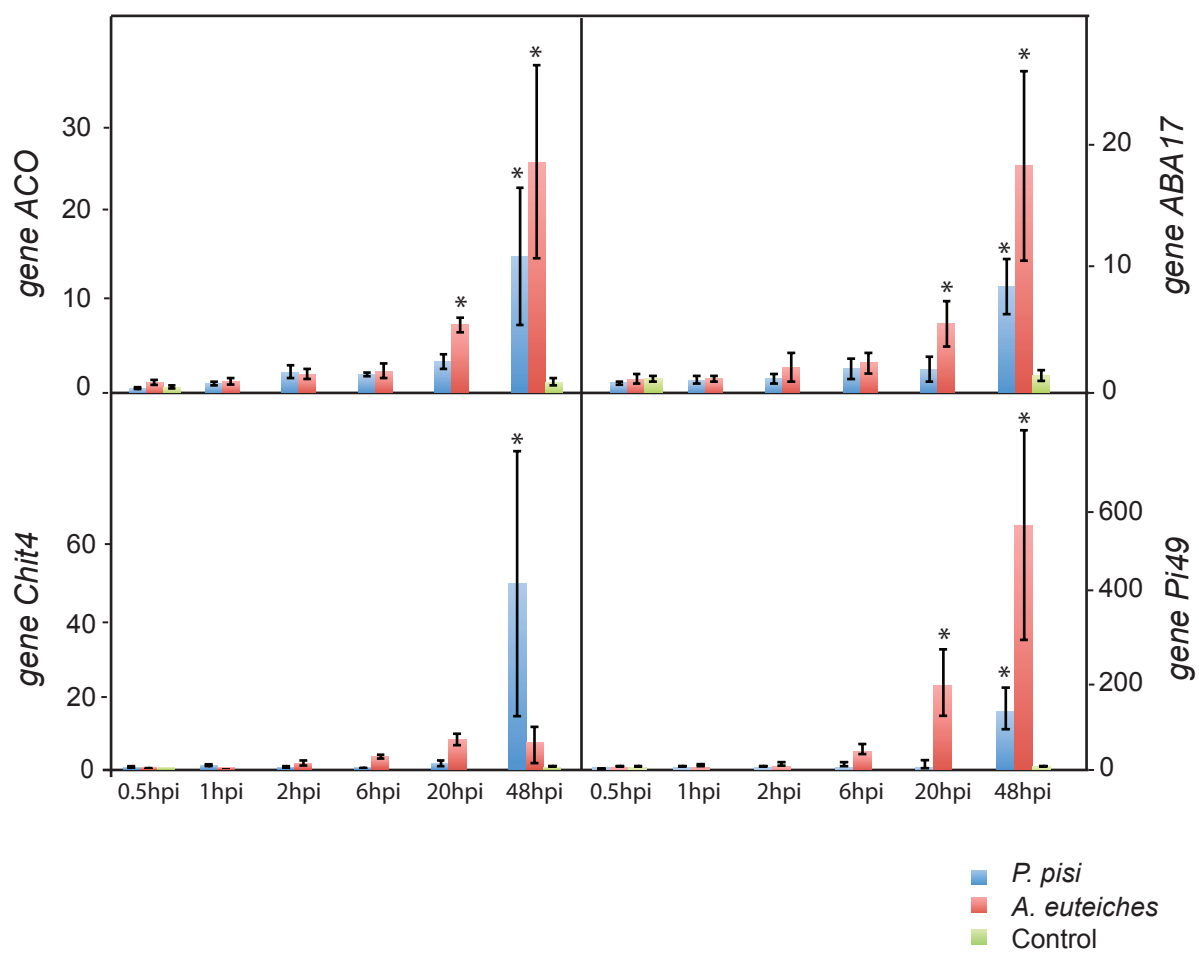

Supplement: Additional file 1: Figure S1. — Expression profiles of selected marker pea genes during interaction with A. euteiches and P. pisi. Expression patterns of four pea genes in response to both pathogens were investigated by RT-qPCR. Relative expression levels were normalized by elongation factor alfa (EFA) expression, and presented in relation to P. pisi-infected plants at 1 hpi (= expression level 1) using the 2-∆∆Ct formula. Error bars represent standard deviation based on three biological replicates. Asterisks indicate statistically significant (P ≤ 0.05) differences between infection treatments and control plants at 48 hpi according to the Fisher test. (PDF 427 kb) [file 12864_2015_1829_MOESM1_ESM.pdf]

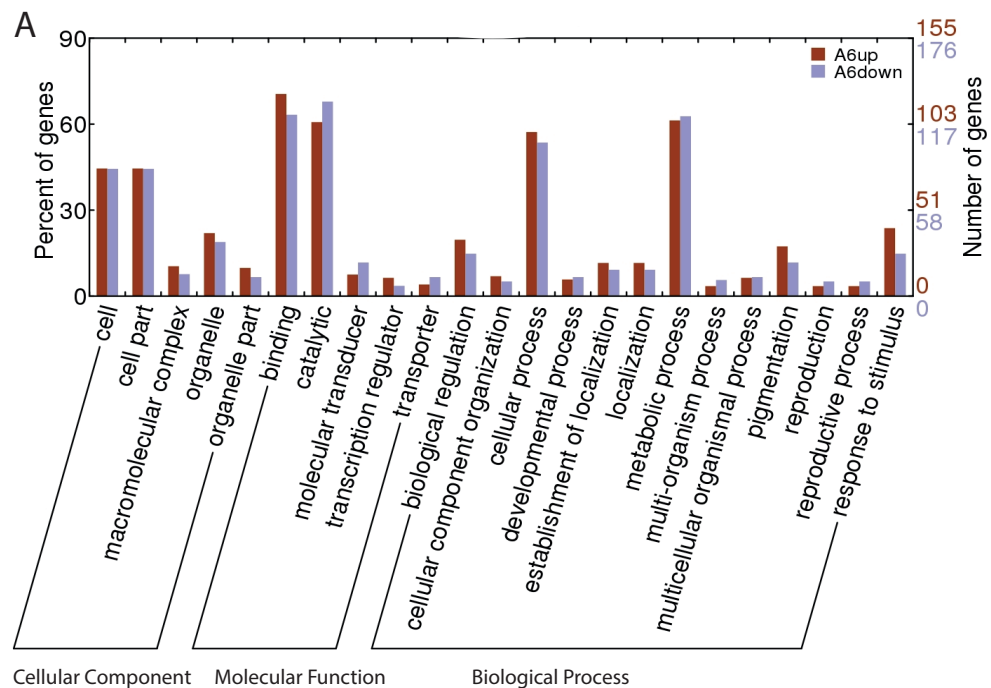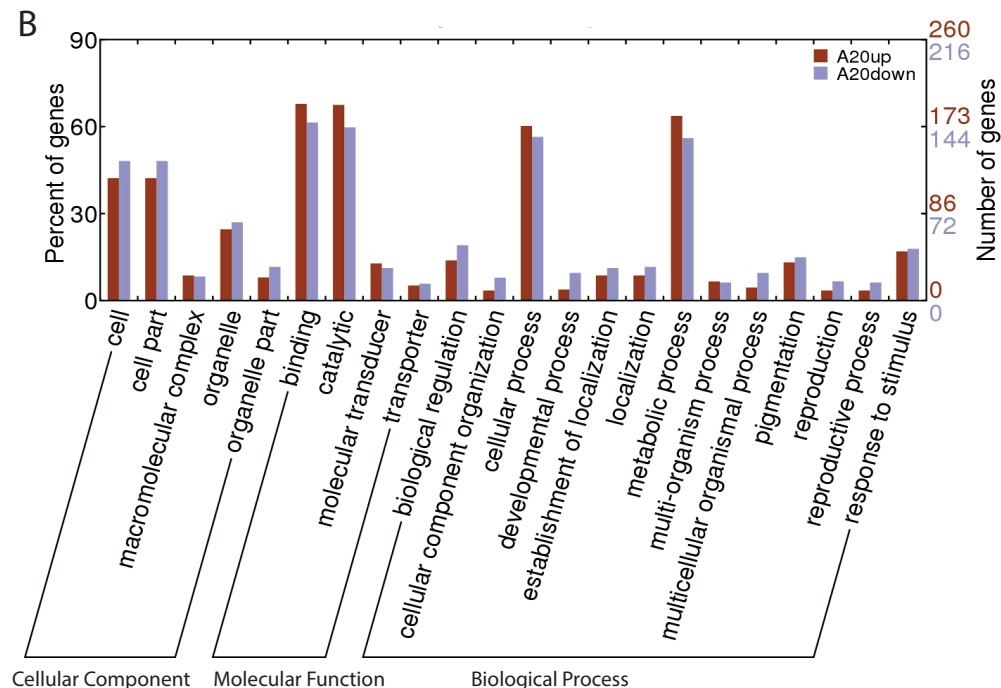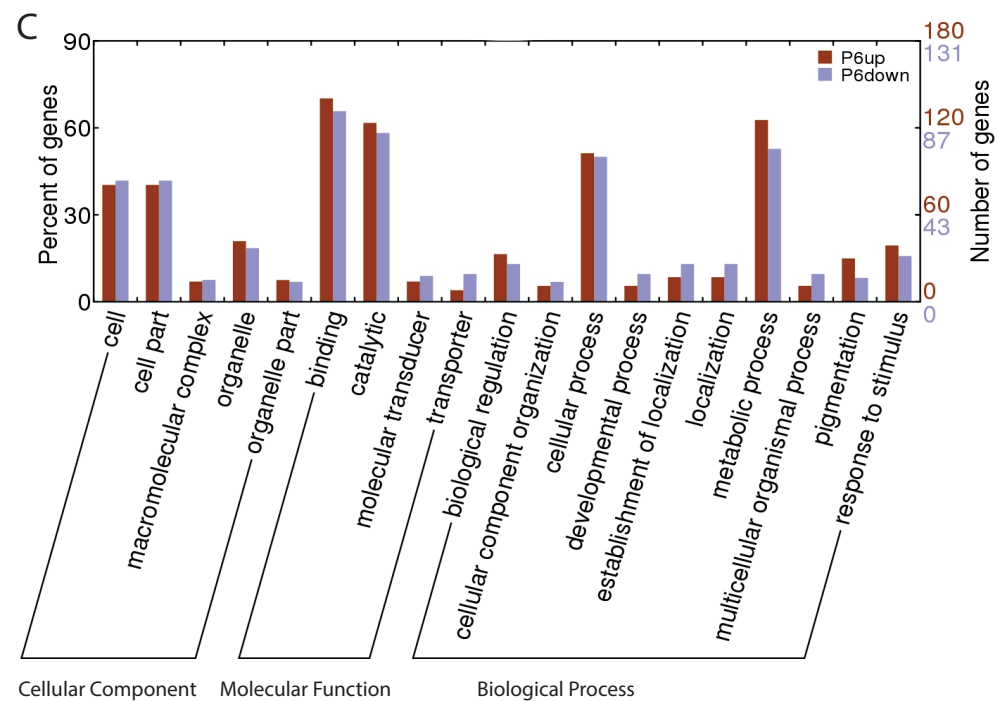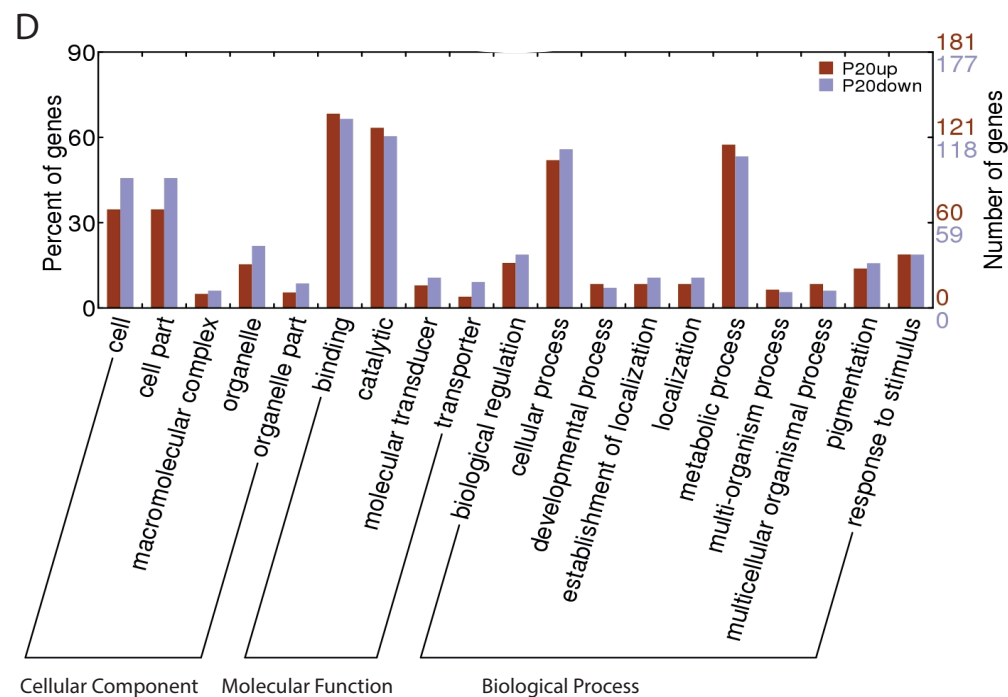

Supplement: Additional file 4: Figure S2. — GO classification of differentially expressed pea genes in response to A. euteiches and P. pisi. Up regulated and down regulated genes (P ≤ 0.05, ≥ 1.5 fold induction or ≤ 0.67 fold repression) in response to A. euteiches at 6 hpi (A) and 20 hpi (B), to P. pisi at 6 hpi (C) and 20 hpi (D). The plots were generated using WEGO. (PDF 2895 kb) [file 12864_2015_1829_MOESM4_ESM.pdf]

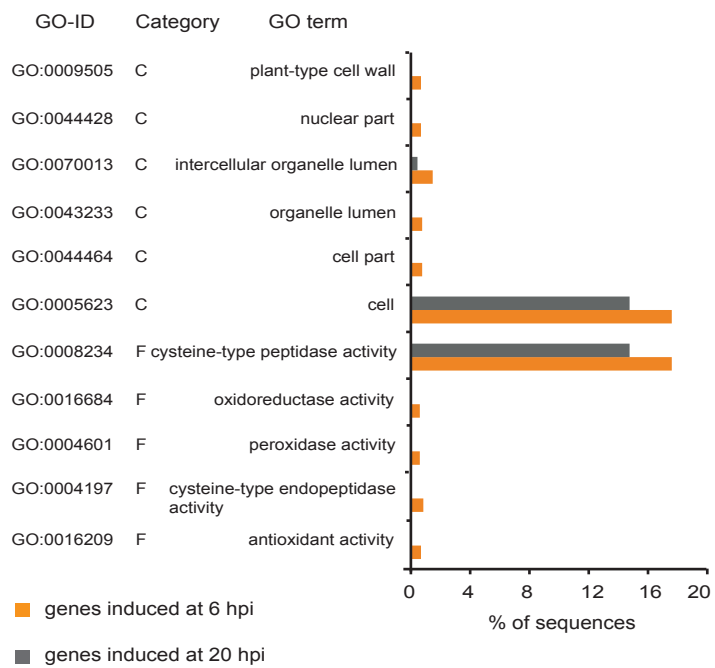

Supplement: Additional file 6: Figure S3. — Gene ontologies (GO) enriched in the early defence transcriptional response in pea. Percentage of genes with significantly enriched (P < 0.005) GO terms that were identified in cluster 1 and 2 (representing genes induced at 6 hpi in response to both pathogens), compared with the background (genes induced at 20 hpi, present in cluster 3 and 4). (PDF 347 kb) [file 12864_2015_1829_MOESM6_ESM.pdf]
