# Supplementary material for: Deciphering common and specific transcriptional immune responses in pea towards the oomycete pathogens Aphanomyces euteiches and Phytophthora pisi
Source: BMC Genomics. 2015 Aug 21;16(1):627. doi: 10.1186/s12864-015-1829-1 (PMC4546216; doi:10.1186/s12864-015-1829-1)
Supplement: Additional file 8: Table S5. — Primers used in this study for RT-qPCR analysis. (DOCX 20 kb) [file 12864_2015_1829_MOESM8_ESM.docx]

**Table S4.** Primers used for RT-qPCR in this study.

| **Pea accession ^a^** | **Medicago Accession ^b^** | **Description ^c^** | **Forward primer** | **Reverse primer** | **Amplicon**  **(bp)** | **Reference** |
| --- | --- | --- | --- | --- | --- | --- |
| Contig, 3065 | AC146575_11.5 | *Chalcone synthase (CHS1)* | ATGCCCGGAGCCGATTACCA | AGCGCCTTTGTTGTTCTCAGCC | 144 |  |
| Contig, 3066 | AC146575_16.5 | *Chalcone synthase (CHS2)* | GCCCGGAGCCGATTACCAACT | TGTCACTAGGGCCACGGAATGT | 200 |  |
| Contig, 3647 | AC155803_41.5 | *Callose synthase (GSL1)* | AATTCAAAACCGCGCTTCTTCAAC | CATCTCACGCGCATCACTTTTCTT | 89 |  |
| Contig, 27779 | AC149580_19.5 | *Lipoxygenase (LOX1)* | CTCAAAGCCCTAGTGGTATCATCG | CAAGAAAGAGGTGGCGGTATCAAT | 89 |  |
| Contig 30903 | AC144516_33.5 | *Peroxidase (POD1)* | GATTGCTTTGTCAGGGGATGTG | TGGATTGTTTGCAGGATGGTCTCT | 90 |  |
| AF369886.1 | AC146632_64.5 | *Resistance protein (NB-LRR1)* | TCGAGGCTTTCCATTATTGTTTTA | GATATCAGATGGCTCCACTTTGTA | 144 |  |
| Contig, 10056 | AC139852_38.4 | *Isoflavonoid-O-methyltransferase (OMT)* | TTGGTGGTGGAACTGGAACA | GCAATCCTCATCACTCCAATCA | 209 |  |
| U69554.1 | AY942158.1 | *6a-hydroxymaackiain methyltransferase (HMM6)* | TTTGAACTTTGTTGGTGGAGATATG | AATCATGCAGAACCCACTTGAGT |  | [14] |
| M98357 | XM_003594113.1 | *Aminocyclopropane-carboxylate oxidase (ACO)* | TTTCTTTTTGCGCCATCTTCC | TTCAACGCAAATTCCTTCATCAC | 93 |  |
|  | AY490790.1 | *Chitinase (Chit4)* | GGTGATGCATATTGTGGCACAGGG | GCAGCAGCAACCTCACGTTTGGAG |  | [31] |
| Z15128 | XM_003594801.1 | *Abscisic acid-responsive protein (ABA17)* | GTCCAGGAACCATCAAGAAGC | AGCCCTGGTCCTCCTACTAAG | 127 |  |
| X13383.1 | XM_003594786.1 | *Disease resistance response (Pi49)* | AAATCACTTCTGTTGTAGCACCTG | TCCTTCAACAATTTCGATACTTTT | 113 |  |

^a^ Pea accession refers to pea gene accession number in GenBank or the contig number in pea transcriptome database by Franssen et al., 2011 [8].

^b^ Indicates the accession number of possible orthologous genes in *M. truncatula* genome.

^c^ The genes *ACO*, *Chit4*, *ABA17* and *Pi49* were used as marker genes for evaluating the infection process.
